# Treatment of Parturition-Induced Rupture of Pubic Symphysis after Spontaneous Vaginal Delivery

**DOI:** 10.1155/2014/485916

**Published:** 2014-01-15

**Authors:** C. Gräf, R. M. Sellei, S. Schrading, D. O. Bauerschlag

**Affiliations:** ^1^Department of Gynecology and Obstetrics, University Hospital RWTH Aachen, 52074 Aachen, Germany; ^2^Department of Orthopaedic Trauma, University Hospital RWTH Aachen, 52074 Aachen, Germany; ^3^Department of Diagnostic and Interventional Radiology, University Hospital RWTH Aachen, 52074 Aachen, Germany

## Abstract

Parturition-induced rupture of pubic symphysis is an uncommon but severe complication of delivery. Characteristic symptoms are an immediate onset of suprapubic and/or sacroiliac pain within the first 24 hours postpartum, often accompanied by an audible crack. Diagnosis can be confirmed by imaging including X-ray, Magnet Resonance Imaging (MRI), and ultrasound. However, there is no consensus on the optimal therapy. Conservative treatment is predominantly used. It has been reported that, in cases of extreme symphyseal rupture with pelvic instability or persisting pain after conservative therapy, operative treatment achieves a successful outcome. In this report, we present a case of a twenty-year-old primigravida who developed suprapubic pain after a nonoperative vaginal birth with shoulder dystocia. A rupture of pubic symphysis with a gap of 60 mm was confirmed by means of X-ray and MRI. Simultaneously, other pelvic joint injuries could be excluded. Operative treatment by an open reduction and internal plate fixation yielded excellent results.

## 1. Introduction

Rupture of pubic symphysis is an uncommon event after vaginal delivery. Reported incidence varies from 1 in 300 to 1 in 30.000 deliveries [1]. While a mild diastasis of the pubic symphysis (i.e., less than 10 mm) is considered to be physiological in pregnancy, greater separation can lead to tenderness of palpation and disability to ambulate [2]. Factors that contribute to a rupture of pubic symphysis are rarely defined. Nevertheless, it seems clear that multiparity, macrosomia accompanied by cephalopelvic disorder, McRoberts maneuver, forceps, maternal connective tissue disorders, prior pelvic trauma, and hyperflected legs may predispose to pubic symphysis diastasis [2–4].

Diagnosis can be confirmed rapidly by pelvic X-ray. Additionally, MRI serves to exclude soft tissue injury. However, there is no consensus on the optimal therapy [5, 6]. Typically, a conservative treatment is performed comprising pelvic girdle, analgesia, bed rest in lateral decubitus, and physical therapy [1, 2, 7–12]. In cases of extreme pubic symphyseal rupture with pelvic instability or persistent pain after conservative therapy, operative treatment is a successful alternative method, which has been reported in several cases [4, 6, 13–15].

## 2. Case Report

**Figure 1:**
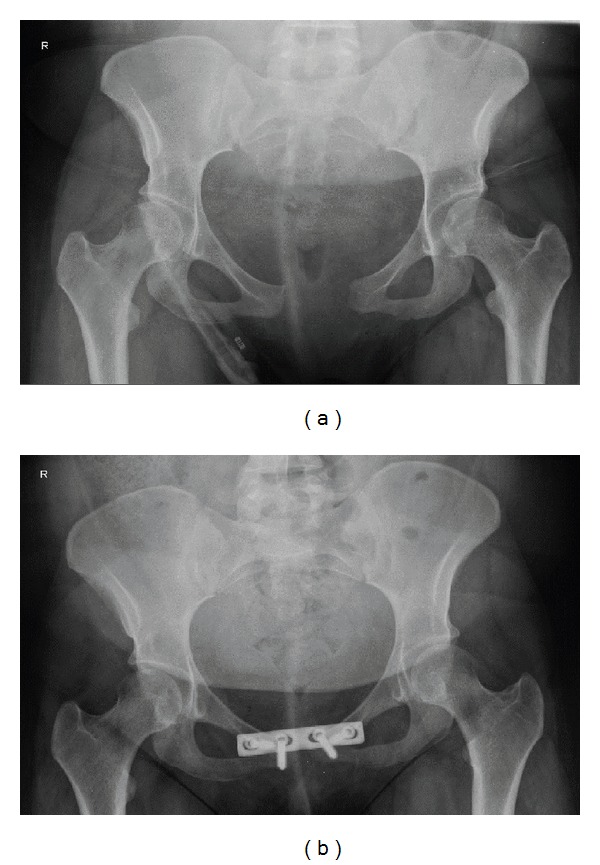
X-ray of the pelvis with a pubic symphysis separation is shown in (a). X-ray of the pelvis after surgical fixation of the symphysis is shown in (b).

A twenty-year-old gravida 1, para 1 was referred to our tertiary care hospital with immediate pain in pubic symphysis on the first postpartum day. The patient had no previous medical or surgical history. Her antenatal course had been uncomplicated. Three days before term, the patient had entered the extern hospital with endogen uterine contractions. After a normal progression of labour, a shoulder dystocia occurred. By performing mediolateral episiotomy, McRoberts maneuver, and suprasymphyseal manual pressure, a girl was delivered. The newborn had a birthweight of 3830 g, a length of 48 cm, and a cranial circumference of 34.8 cm. APGAR values were 9/9/10 and the arterial pH was measured to be 7.12. The newborn presented an extremely swollen right arm which required a transfer to the neonatology department of our tertiary care hospital. It was later diagnosed as a hemangioendothelioma of the right arm. On the first postpartum day, the mother developed strong suprasymphyseal pain that appeared after ambulating and was therefore transferred to our tertiary care hospital. On the physical examination the patient had a painful and palpable dehiscence of the pubic symphysis. Pelvic horizontal instability was identified but no sign of vertical instability. There were no symptoms of active bleeding or lesions of urinary tract or neurologic deficits. In addition, a pelvic X-ray revealed a pubic symphysis separation of 60 mm. This gap is shown in Figure 1(a).

**Figure 2:**
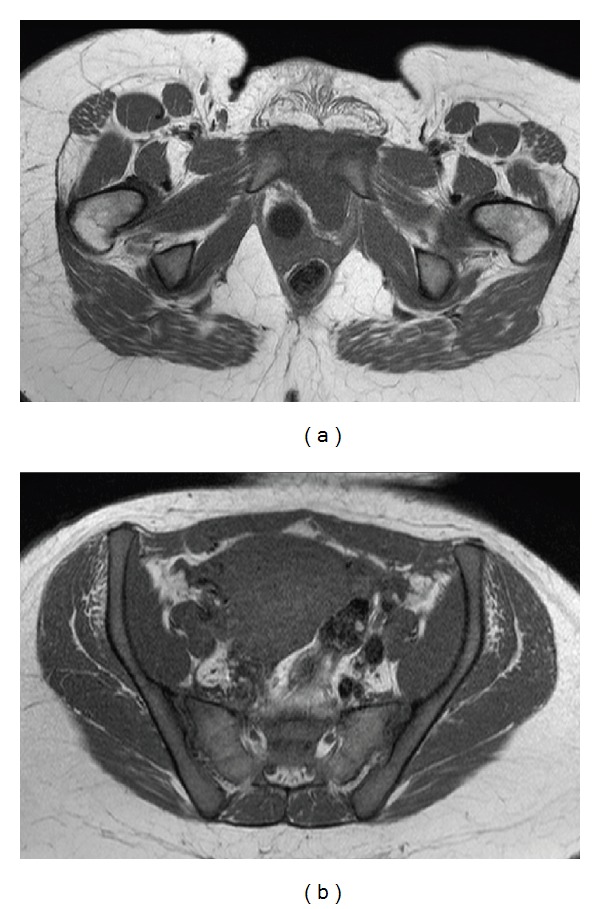
Axial T1-weighted images without contrast are shown in (a) and (b). (a) shows the MRI that confirms the pubic symphysis separation. No separation of the iliosacral joints and no pelvic fracture can be identified in (b).

**Figure 3:**
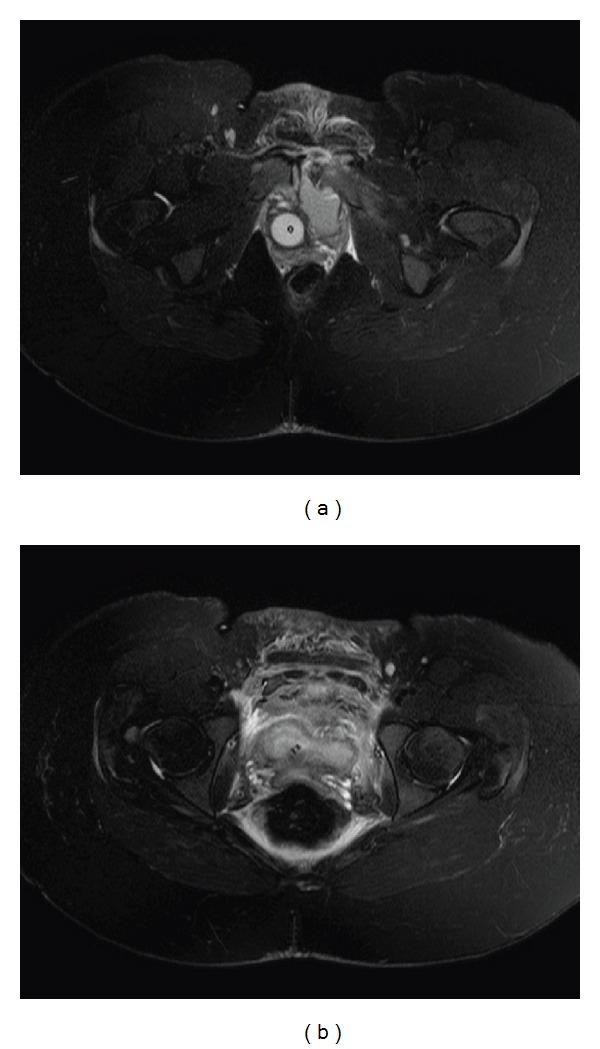
Axial T2-weighted images with fat-saturation (SPIR) are shown in (a) and (b). In addition, a hematoma around the symphysis pubis is shown, which extends to the small pelvis.

The MRI, shown in Figures 2(a) and 2(b), confirmed a single pubis symphysis rupture with no lesions of the sacroiliac joints. Moreover, a hematoma around the symphysis pubis was discovered, however, without evidence of tissue injury. The hematoma can be seen in Figures 3(a) and 3(b). Starting therapy with a pelvic binder, bed rest, and analgesia, the patient underwent an open reduction and internal fixation by means of a plate on the sixth postpartum day. The patient received physical therapy to ambulate and was discharged on the fifth postoperative day. After 2 weeks the patient was able to ambulate without complaints and to take care of her child. A postoperative radiographic control determined the correct position of the implant, which can be seen in Figure 1(b). This was again confirmed three months later.

## 3. Discussion

Although the initial clinical examination and diagnostic investigation are straightforward, the optimal way of treating a peripartum pubic symphysis rupture is discussed controversially. Several reports have shown that a conservative therapy is a reasonable approach [1, 2, 9–12]. Even in cases of large symphyseal ruptures measuring 5 cm [8] and 9 cm including iliosacral jointrupture [7], a successful conservative therapy has been reported. However, other works have demonstrated the limitations of a conservative treatment. For instance, Kharrazi et al. [4] presented four cases of pelvic and sacroiliac joint rupture after vaginal birth; in those women undergoing conservative therapy, posterior pelvic pain remained for more than two years. In addition, Rommens [14] reported three cases of postpartum pubic symphysis rupture with persisting pain after conservative therapy. Those patients did not recover completely until they were operated by an open reduction and internal plate fixation. Niederhauser et al. [3] demonstrated a similar case; after a symphyseal rupture of 60 mm occurring in a spontaneous vaginal birth with shoulder dystocia, conservative treatment failed to provide an optimal outcome. A 25 mm gap was still present after 3 months and pain also persisted. Finally, surgical treatment by means of an open reduction and internal fixation yielded optimal results. Chang and Wu [15] showed that, in case of contraindication of a plate fixation due to a contaminated pelvic environment, an external fixation can be an equivalent surgical method of pubic symphysis diastasis. Dunivan et al. [6] also underlined the advantages of an immediate external fixation in a case of a gap of pubic symphysis measuring 62 mm.

As a consequence, these works suggest the indication of an operative approach if a gap of the pubic symphysis is larger than 40 mm [4, 6, 13, 14]. As we highlight in our case report, we agree with this threshold.

## 4. Conclusions

Pubic symphysis rupture is an uncommon but often underestimated injury after vaginal delivery that can lead to significant chronic disability. Therefore, in case of peripartum suprapubic pain, it is important to consider a pubic symphyseal diastasis that requires interdisciplinary treatment. In cases of a gap greater than 40 mm, a surgical intervention may result in better outcome including shorter hospitalization, earlier ambulation, and the opportunity to cope with the new circumstances of her motherhood.
